# Genome Sequence of a Novel Strain of a Phasi Charoen-Like Virus Identified in Zhanjiang, China

**DOI:** 10.1128/genomeA.01024-17

**Published:** 2018-01-11

**Authors:** Xiaomin Zhang, Tao Jin, Yalan Huang, Suibin Huang, Fan Yang, Peng Lin, Yang Liu, Dana Huang, Chunli Wu, Jianbin Xie, Jinquan Cheng, Chengsong Wan, Renli Zhang

**Affiliations:** a Shenzhen Center for Disease Control and Prevention, Shenzhen, China; b School of Public Health and Tropical Medicine, Southern Medical University, Guangzhou, China; c China National Genebank–Shenzhen, BGI-Shenzhen, Shenzhen, China; d BGI Education Center, University of Chinese Academy of Sciences, Shenzhen, China

## Abstract

Here, we report the genome sequence of a novel strain of a Phasi Charoen-like virus, isolated from *Aedes aegypti* mosquitoes caught in Zhanjiang province of China. Phylogenetic analysis suggested that this virus belongs to a new genus, *Goukovirus*, in the family *Bunyaviridae*. This is the first reported genome sequence of a Phasi Charoen-like virus identified in China.

## GENOME ANNOUNCEMENT

The family *Bunyaviridae* is one of the most diversified families of RNA viruses (1). It is traditionally characterized into five genera, *Nairovirus*, *Orthobunyavirus*, *Phlebovirus*, *Hantavirus*, and *Tospovirus* (1). Recently, several novel insect-specific bunyaviruses have been identified from *Aedes aegypti* or *Culex* mosquitoes in Southeast Asia and Africa (2–7). Of note, all of these viruses showed a genome-encoding strategy distinct from other traditional bunyaviruses and could infect only mosquito cells rather than vertebrate cells. Therefore, these newly discovered viruses were proposed to be classified to a novel genus, *Goukovirus*, of the family *Bunyaviridae* (7).

The *A. aegypti* mosquito is an important vector for transmitting many pathogenic arboviruses, such as dengue virus, chikungunya virus, and Zika virus (3). Investigation of novel viruses in *A. aegypti* mosquitoes is of great value, as it can facilitate our understanding of viral diversity and provide awareness of potential arthropod-borne pathogens. To identify whether there is any novel virus in wild *A. aegypti* mosquitoes, we collected larvae from small water bodies in scrape tires and water tanks in the town of Wushi in Zhanjiang province, China. After the emergence of adult mosquitoes, total RNA was extracted from the homogenate of pooled female *A. aegypti* mosquitoes using a Qiagen RNeasy minikit (Qiagen). Then, the RNA sample was sent to the Beijing Genome Institute (BGI) for deep sequencing. A BLASTn search against the NCBI nucleotide (nt) database determined that the most abundant sequences assembled were three genome segments of a Phasi Charoen-like virus; this was further confirmed by Sanger sequencing of both pooled female and male *A. aegypti* RNA samples.

This virus was tentatively named Zhanjiang01 according to its isolated source. Similar to other Phasi Charoen-like viruses, the nearly complete genome sequence of the Zhanjiang01 virus consisted of three single-stranded RNA segments of 6,773 nt (segment L), 3,840 nt (segment M), and 1,333 nt (segment S), encoding a viral RNA-dependent RNA polymerase (2,217 amino acids [aa]), a glycoprotein precursor (1,237 aa), and a nucleocapsid protein (268 aa), respectively. Interestingly, the nonstructural (NS) protein-coding gene, which was absent in the genes of other insect-specific viruses, was not found in the M and S segments. Pairwise nucleotide identities among all the Phasi Charoen-like viruses ranged from 95% to 97%, while the aa identities ranged from 97% to 98%. Phylogeny based on both the nt and aa sequences indicated that the Zhanjiang01 virus was in the clade of the recently proposed novel genus *Goukovirus* within the family *Bunyaviridae*. The detection of the same genome sequences in male adult mosquitoes suggests that this virus might be vertically transmitted.

To our knowledge, this is the first report of a Phasi Charoen-like virus identified from *A. aegypti* in China. This discovery further enhances our understanding of the wide distribution and high diversity of insect-specific bunyaviruses and flaviviruses. In particular, the potential use of the Zhanjiang01 isolate as a vaccine is worthy of further study.

### Accession number(s).

Genome data for the Phasi Charoen-like virus isolate Zhanjiang01 have been deposited in GenBank under the accession numbers MF614132 (segment L), MF614133 (segment M), and MF614134 (segment S).
